# Ethanol-Enriched Substrate Facilitates Ambrosia Beetle Fungi, but Inhibits Their Pathogens and Fungal Symbionts of Bark Beetles

**DOI:** 10.3389/fmicb.2020.590111

**Published:** 2021-01-13

**Authors:** Maximilian Lehenberger, Markus Benkert, Peter H. W. Biedermann

**Affiliations:** ^1^Research Group Insect-Fungus Symbiosis, Department of Animal Ecology and Tropical Biology, University of Würzburg, Würzburg, Germany; ^2^Chair of Forest Entomology and Protection, University of Freiburg, Freiburg im Breisgau, Germany

**Keywords:** ambrosia fungi, bark and ambrosia beetles, symbiont selection, ethanol, detoxification, *Ips typographus*

## Abstract

Bark beetles (*sensu lato*) colonize woody tissues like phloem or xylem and are associated with a broad range of micro-organisms. Specific fungi in the ascomycete orders Hypocreales, Microascales and Ophistomatales as well as the basidiomycete Russulales have been found to be of high importance for successful tree colonization and reproduction in many species. While fungal mutualisms are facultative for most phloem-colonizing bark beetles (*sensu stricto*), xylem-colonizing ambrosia beetles are long known to obligatorily depend on mutualistic fungi for nutrition of adults and larvae. Recently, a defensive role of fungal mutualists for their ambrosia beetle hosts was revealed: Few tested mutualists outcompeted other beetle-antagonistic fungi by their ability to produce, detoxify and metabolize ethanol, which is naturally occurring in stressed and/or dying trees that many ambrosia beetle species preferentially colonize. Here, we aim to test (i) how widespread beneficial effects of ethanol are among the independently evolved lineages of ambrosia beetle fungal mutualists and (ii) whether it is also present in common fungal symbionts of two bark beetle species (*Ips typographus*, *Dendroctonus ponderosae*) and some general fungal antagonists of bark and ambrosia beetle species. The majority of mutualistic ambrosia beetle fungi tested benefited (or at least were not harmed) by the presence of ethanol in terms of growth parameters (e.g., biomass), whereas fungal antagonists were inhibited. This confirms the competitive advantage of nutritional mutualists in the beetle’s preferred, ethanol-containing host material. Even though most bark beetle fungi are found in the same phylogenetic lineages and ancestral to the ambrosia beetle (*sensu stricto*) fungi, most of them were highly negatively affected by ethanol and only a nutritional mutualist of *Dendroctonus ponderosae* benefited, however. This suggests that ethanol tolerance is a derived trait in nutritional fungal mutualists, particularly in ambrosia beetles that show cooperative farming of their fungi.

## Introduction

Filamentous fungi are generally known to be common symbionts of bark and ambrosia beetles (Curculionidae: Scolytinae and Platypodinae). While many species use beetles for dissemination with apparently little benefits for their vectors (Birkemoe et al., 2018; Seibold et al., 2019) other fungi are essential for the beetles like nutritional or tree-defenses-detoxifying mutualists as well as tree-defenses-stimulating fungi (e.g., Six, 2003; Lieutier et al., 2009; Kandasamy et al., 2019; Biedermann and Vega, 2020). The most famous nutritional mutualisms are those between xylem-colonizing ambrosia beetles and various “ambrosia fungus” species in the ascomycete orders Hypocreales, Microascales and Ophiostomatales as well as phloem-colonizing *Dendroctonus* bark beetles and their basidiomycete and ascomycete mutualists in the orders Russulales and Ophiostomatales (Barras and Perry, 1972; Six and Paine, 1998; Six and Klepzig, 2004; Harrington, 2005; Kirkendall et al., 2015; Biedermann and Vega, 2020). All these are truly agricultural mutualisms, because they evolve active care of the beetles for their fungal “crops” and involve more or less species-specific partnerships. Indeed, within the majority of the ambrosia beetle – fungus relationships, the beetles are essential for the survival of their mutualistic fungi as they would be overgrown by fungal competitors if the beetles are not present (Kirkendall et al., 2015; Nuotclà et al., 2019). Up to date, these mutualisms are unique for beetles and comparable to the advanced fungi culturing systems of attine ants and fungus-farming termites (Farrell et al., 2001; Six, 2003; Müller et al., 2005; Biedermann and Vega, 2020).

The nutritional mutualisms of ambrosia beetles and certain *Dendroctonus* spp. as well as some *Ips* sp. are known for quite a while (Batra, 1963; Francke-Grosmann, 1965, 1966, 1967; Six, 2003; Vissa and Hofstetter, 2017), but more recent research shows that other bark beetle species also depend on fungal associates mainly through detoxification of insect-repelling tree-chemistry (like in the case of fungal symbionts of the European spruce bark beetle *Ips typographus*; Kandasamy et al., 2016, 2019; Wadke et al., 2016; Zhao et al., 2019). While nutritionally important fungal mutualists are typically vertically transmitted within mycetangia (i.e., fungus spore carrying and selecting organs), these organs are rather rare in non-nutritional beetle-fungus associations (Francke-Grosmann, 1956, 1967; Batra, 1963; Six, 2003; Hulcr and Stelinski, 2017; Skelton et al., 2020; Biedermann and Vega, 2020). How mutualisms are maintained in the latter case is poorly known and supposedly involves vertical transmission through the gut or the surface of the exoskeleton of the insects (Six, 2003; Harrington, 2005). Even less understood is how the beetles maintain the dominance of certain fungi within their tunnel systems and how they suppress ubiquitous competitor fungi and beetle pathogens (henceforth termed *antagonistic fungi*).

So far, three evolutionary mechanisms are known by which a host (e.g., a bark beetle) can maintain a mutualism with a beneficial symbiont (e.g., an ambrosia fungus): (i) Partner choice, in which a host is actively selecting a specific symbiont, (ii) partner fidelity, where symbionts are vertically transmitted from one host generation to the next, and (iii) the theoretical and empirically hardly studied mechanism of competition-based screening, in which environmental filters created by the host select for the preferred symbiont (Archetti et al., 2011; Scheuring and Yu, 2012; Foster et al., 2017). Environmental screening of mutualistic ambrosia fungi utilizing ethanol within woody substrate (that most ambrosia beetles preferentially colonize) has been recently discovered by Ranger et al. (2018). These authors show that ethanol strengthens the competitive ability of mutualistic ambrosia fungi over other fungal antagonists, because ambrosia fungi are able to detoxify ethanol and use it as a carbon source, whereas the antagonists are strongly inhibited in their growth by even small amounts of ethanol, which is typically an antimicrobial compound (McGovern et al., 2004; Tunc et al., 2007). Moreover, mutualistic ambrosia beetle fungi are known to produce ethanol and other alcohols themselves (Kuhns et al., 2014; Kandasamy et al., 2016), giving them the possibility to enrich the colonized woody substrate with ethanol and thus maintain their dominance even after the production by the dying plant cells ceases (Kimmerer and Kozlowski, 1982). The ethanol production of ambrosia fungi can thus be compared with other similar defensive mechanisms used by other microorganisms to protect themselves and their animal hosts (Cardoza et al., 2006; Scott et al., 2008; Six, 2013; Flórez et al., 2015; Ranger et al., 2018).

Ethanol generally plays a crucial role for the attraction of ambrosia beetles (i.e., it is a kairomone) as it allows them to detect suitable hosts like stressed or recently dead trees (Graham, 1968; Kühnholz et al., 2001; Ranger et al., 2015). In fact, ethanol is not solely present in stressed trees but is commonly found also in healthy trees within both xylem and phloem (Kimmerer and Stringer, 1988; MacDonald and Kimmerer, 1991; Kozlowski, 1992; Kelsey et al., 2014). Its content is known to increase as soon as the tree is stressed (e.g., flood stress, mechanical damage) (Kimmerer and Kozlowski, 1982; MacDonald and Kimmerer, 1991; Ranger et al., 2013) and is thereby functioning as a cue for ambrosia beetles to recognize defense deficient trees. The phloem-colonizing bark beetles, which are the evolutionary ancestors of ambrosia beetles, still prefer similarly deficient host trees. On the contrary, however, they usually use tree volatiles and/or aggregation pheromones (produced *de novo*) other than ethanol to detect their preferred hosts (Vité et al., 1972; Wood, 1982; Kirkendall et al., 2015; Kandasamy et al., 2016; Biedermann et al., 2019). So currently it is unknown whether ethanol plays a role in the bark beetle system by preferentially fostering the growth of their fungal mutualists.

Ambrosia beetle fungi are not unique in their ability to produce ethanol. In fact, the ability of yeasts and filamentous fungi to produce ethanol is relatively widespread in certain fungal groups (e.g., plant-colonizing fungi like *Fusarium* and *Rhizopus*; the brewer’s yeast *Saccharomyces cerevisiae*; saprobic and soil-colonizing fungi like *Aspergillus*) (Schneider and Jeffries, 1989; Singh and Kumar, 1991; Singh et al., 1992; Wainwright et al., 1994; Skory et al., 1997; Ferreira et al., 2014) and has an important role in biotechnologically used fermentation processes. While many of these taxa are able to create their own defended niche by metabolizing, producing and accumulating ethanol in their environment, they are typically found free-living and not in mutualisms with insect hosts (Thomson et al., 2005; Dashko et al., 2014; Zhou et al., 2017). The only other insects known to be associated with an ethanol-producing fungus (i.e., yeast) are *Drosophila* species, which benefit, like the ambrosia beetles, from the defensive abilities of their symbionts (McKenzie and Parsons, 1972; Becher et al., 2012; Christiaens et al., 2014). Some fungal mutualists of bark beetles like *Endoconidiophora polonica* and *Grosmannia penicillata* (associated with *Ips typographus*), are as well known to produce several different alcohols (Kandasamy et al., 2019), but whether they metabolize, produce and use them to defend themselves like the ambrosia beetle mutualists remains unstudied.

Nutritional fungus mutualisms have evolved independently in at least eleven lineages of wood-boring weevils (Coleoptera: Curculionidae: Scolytinae and Platypodinae) with at least five lineages of ascomycetes (Ophiostomataceae, Nectriaceae, Bionectriaceae, Saccharomycetaceae, Ceratocystidaceae) as well as two lineages of basidiomycetes (Peniophoraceae, Meruliaceae) (Alamouti et al., 2009; Li et al., 2014; Hulcr et al., 2015; Bateman et al., 2017; Hulcr and Stelinski, 2017; Biedermann and Vega, 2020). The ancestral habit of these weevils is phloem and most lineages transitioned to colonize xylem after the origin of the nutritional mutualism (which made it possible to live on the nutrient-poor xylem), possibly because of the high competition within the phloem (Kirkendall et al., 2015; Biedermann and Vega, 2020). Currently, only a few ambrosia beetle fungal mutualists in the Ophiostomataceae and Ceratocystidaceae have been investigated for their affinity to ethanol (Ranger et al., 2018) and it remains unknown how widespread this trait is for the other lineages of ambrosia fungi as well as and in particular for the ancestral fungal symbionts of bark beetles.

**FIGURE 1:**
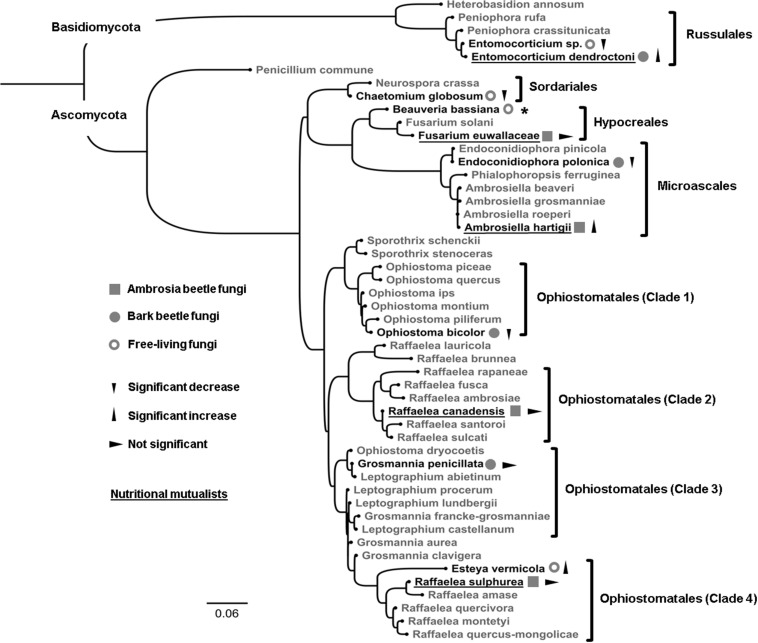
Phylogenetic placement based on 49 fungal LSU sequences. Black-colored species were examined within this study (*N* = 12), while species in gray act as a fungal outgroup (*N* = 37). The LSU region of the following fungi was sequenced by us and used for the phylogenetic analysis (strain IDs in brackets): *Ophiostoma bicolor* (P22), *Grosmannia penicillata* (2), *Endoconidiophora polonica* (F5), *Beauveria bassiana* (P13), *Entomocorticium* sp. (P8), *Entomocorticium dendroctoni* (P163), and *Fusarium euwallaceae* (P170). Sequences from all other species were obtained from NCBI GenBank. For accession numbers and further information of each individual strain see Supplementary Tables 1, 2. Ophiostomatales were divided into their four sub-clades that are currently hypothesized to have evolved independently as nutritional mutualists of ambrosia beetles (Biedermann and Vega, 2020). All examined species were classified into (i) ambrosia beetle fungi, (ii) bark beetle fungi, and (iii) free-living fungi. Triangles denote significant effects on fungal biomass (decrease, increase or not significant; *p* < 0.05) between the 0 and 1% EtOH treatments. Nutritional mutualists of bark and ambrosia beetles are underlined. Due to its abundant sporulation we could not receive comparable data from *Beauveria bassiana* (indicated with *) and excluded it from all further analyses.

Some bark and ambrosia beetles are severe pests, causing high economic damages in forests and plantations worldwide (Kühnholz et al., 2001; Hulcr and Dunn, 2011; La Spina et al., 2013; Ranger et al., 2015; Biedermann et al., 2019). Therefore, research on the physiology of their fungal mutualists is very important to understand the ecology of these beetle-fungus interactions, which may help at some point to develop new management tools that target the fungal mutualists. Here, we examined 10 fungal species from four convergently evolved clades of bark and ambrosia beetle associated fungal symbionts, including five nutritional and two tree-chemistry-detoxifying mutualists and one symbiont with unknown role as well as 2 ubiquitous antagonistic fungi for their ability to grow on ethanol-containing substrate. Two free-living, non-mutualistic filamentous fungi in the same lineages were included to test for phylogenetic effects of ethanol tolerance. Our aims were (i) to test the ethanol tolerance of previously not-tested clades of bark and ambrosia beetle fungal mutualists in the basidiomycete Russulales and the ascomycete Hypocreales and Ophiostomatales (see Figure 1) and (ii) to compare it between bark beetle (*sensu stricto*) and ambrosia beetle fungal partners. The latter can help to understand the evolution of ethanol tolerance because ambrosia beetle fungal mutualists are descendants of bark beetle fungal mutualists, which have not been investigated for their tolerance toward ethanol so far. Finally, we aim to test (iii) how widespread the ethanol sensitivity is in antagonists of bark and ambrosia beetles and free-living fungi in the same clades of known beetle mutualists.

## Materials and Methods

### Fungal Strains

In this study, we used common fungal mutualists/symbionts of widespread bark and ambrosia beetles from the three ascomycete orders [Microascales (Ceratocystidaceae), Ophiostomatales (Ophiostomataceae), Hypocreales (Nectriaceae)] and one basidiomycete order [Russulales (Peniophoraceae)]. Additionally, we used two common fungal antagonists of bark and ambrosia beetles in the two ascomycete orders Hypocreales (Cordycipitaceae) and Sordariales (Chaetomiaceae). Finally, we included the nematophagous fungus *Esteya vermicola* in the Ophiostomatales (Ophiostomataceae) as well as the basidiomycete *Entomocorticium sp.* (see Lehenberger et al., 2018) in the Russulales (Peniophoraceae), which served as non-beetle-associated, free-living, phylogenetic controls (for an overview of all strains see Table 1 and Supplementary Table S1). We included all fungal species within a phylogenetic replacement that aimed to (i) visualize the independently evolved phylogenetic lineages of all tested species and to (ii) indicate each classification as well as (iii) our current findings.

**TABLE 1:** Overview of the twelve fungal isolates used in this study and additional information on their classification, beetle host(s), association, phylogenetic order and literature.

Fungal species	Classification	Beetle host(s)	Association	Phylogenetic order	References
*Entomocorticium* sp.	Free-living	None	None	Russulales	Lehenberger et al., 2018
*Entomocorticium dendroctoni*	Bark Beetle	*Dendroctonus ponderosae*	Nutritional mutualist	Russulales	Whitney et al., 1987
*Endoconidiophora polonica*	Bark Beetle	*Ips typographus*	Tree-defenses-detoxifying mutualist	Microascales	De Beer et al., 2014
*Ophiostoma bicolor*	Bark Beetle	*Ips* spp.	Symbiont with unknown role	Ophiostomatales (Clade 1)	Davidson, 1955
*Grosmannia penicillata*	Bark Beetle	*Ips typographus*	Tree-defenses-detoxifying mutualist	Ophiostomatales (Clade 3)	Goidanich, 1936
*Fusarium euwallaceae*	Ambrosia Beetle	*Euwallacea fornicatus*	Nutritional mutualist	Hypocreales	Freeman et al., 2013
*Ambrosiella hartigii*	Ambrosia Beetle	*Anisandrus dispar*	Nutritional mutualist	Microascales	Batra, 1967
*Raffaelea canadensis*	Ambrosia Beetle	*Xyleborinus saxesenii*	Nutritional mutualist	Ophiostomatales (Clade 2)	Batra, 1967
*Raffaelea sulphurea*	Ambrosia Beetle	*Xyleborinus saxesenii*	Nutritional mutualist	Ophiostomatales (Clade 4)	Harrington et al., 2010
*Chaetomium globosum*	Pathogen	*Various*	Resource competitor	Sordariales	Fries, 1829
*Beauveria bassiana*	Pathogen	*Various*	Entomo-pathogen	Hypocreales	Vuillemin, 1912
*Esteya vermicola*	Free-living	None	None	Ophiostomatales (Clade 4)	Liou et al., 1999

A stock collection of all these fungal isolates in glycerol (80%) and glycerol/peptone (80%/1%; Roth, Germany) is constantly maintained at −80°C in our lab in Freiburg, Germany. Origins of the fungal isolates are given in Supplementary Table 1. At the beginning of the experiment, we first revived fungi on malt extract agar plates (MEA: 3% malt extract, 0.5% soy peptone, 2% agar, pH = 5.5–6; Sigma-Aldrich, Germany) and stored them at 5°C until they were sub-cultured once more on MEA (25°C, 60% RH) for 5 (fast growers) to 14 (slow growers) days (Supplementary Table 3) until the experiment was started for each fungus individually.

### DNA Extraction and LSU/βT/ITS Barcoding

We first homogenized fungal samples (pure biomass) by grinding in liquid nitrogen and then proceeded with DNA extraction using the NORGEN Biotek Corp. fungi/yeast genomic DNA isolation kit following the manufacturer’s protocol. Sequences of the large subunit (LSU) of the ribosomal RNA gene were primarily used for the identification and phylogenetic placement of isolates (Figure 1; Supplementary Table 1). For LSU amplification, we used the common primers LROR-F (GTACCCGCTGAACTTAAGC) and LR5-R (ATCCTGAGGGAAACTTCG) (Vilgalys and Hester, 1990; Rehner and Samuels, 1994), which amplify a region of approximately 830 to 880 bp.

Because some samples repeatedly failed to amplify using the LSU primers, we barcoded them using the internal transcribed spacer (ITS) of the ribosomal RNA or the beta-tubulin gene (βT). The ITS region was amplified using primers ITS1-F (TCCGTAGGTGAACCTGCGG) and ITS4-R (TCCTCCGCTTATTGATATGC) (White et al., 1990) and with an annealing temperature of 54.5°C. The ITS region finally contained approximately 580 bp. For amplification of βT, we used the primers T10-F (ACGATAGGTTCACCTCCAGAC) and Bt2b-R (ACCCTCAGTGTAGTGACCCTTGGC) (Glass and Donaldson, 1995; O’Donnell and Cigelnik, 1997) and 57.5°C as annealing temperature. Sequence lengths contained around 300 to 400 bp.

For all PCR reactions, we used a similar master-mix for LSU, βT and ITS barcoding [Master-mix for 50 μl: 25 μl 2× phusion high-fidelity PCR master mix with GC buffer (Thermo Scientific^TM^, Germany), 2.5 μl forward primer 1 (10 μM, Eurofins Genomics, Germany) 2.5 μl reverse primer 2 (10 μM, Eurofins Genomics, Germany), 18 μl ddH20, 2 μl template (usually 1:10 diluted)]. For all reactions we applied the following PCR conditions: 98°C for 30 s, followed by 35 cycles at 98°C for 10 s, 55.5°C, 54.5°C or 57.5°C (for LSU, ITS, and βT, respectively) for 30 s and 72°C for 20 s, ending with 72°C for 10 min and ending with a storage temperature of 5°C.

DNA-purification was done using the Wizard^®^ SV gel and PCR clean-up system (Promega, Germany) after performing gel electrophoresis. Sanger-sequencing was performed by Eurofins (Eurofins Genomics, Germany). To verify each sequence quality, we used the “SnapGene^®^ Viewer” 3.2 (SnapGene software, from GSL Biotech; available at snapgene.com) and manually corrected automatically deduced sequences, if necessary. Fungal species were finally identified using BLASTn at NCBI (Altschul et al., 1990). All obtained fungal sequences were uploaded to the NCBI GenBank database (the accession numbers are included in Supplementary Table 1).

### Phylogenetic Analysis

Sequences of the LSU for seven of our fungal sequences were used for maximum likelihood analyses and construction of a phylogenetic tree (Supplementary Table 1). NCBI GenBank entries were used for the remaining five sequences of studied fungi as well as for 37 fungal outgroup sequences (Supplementary Table 2). Individual sequences were prepared using GeneDoc 2.7 (Nicholas, 1997) or MEGA 7.0 (Kumar et al., 2016) and were additionally compared with the received output from Clustal Omega (Madeira et al., 2019). The phylogenetic analyses were done with the IQ-Tree-Web-Server (Nguyen et al., 2015; Trifinopoulos et al., 2016) using the ultrafast bootstrap analysis (Minh et al., 2013). Based on the web server FindModel (Posada and Crandall, 1998, available at^1^), we chose the generalized time reversible (GTR) model plus gamma (+ G) with rate heterogeneity (rate categories – 4) and combined it with 8.000 bootstrap alignments as our substitution model. FigTree^2^ was used for visualization of the phylogenetic tree.

The phylogenetic relationship of tested fungi is already known (see Rollins et al., 2001; Alamouti et al., 2009; Dreaden et al., 2014; Vanderpool et al., 2018), therefore we did not include it within the results. The aim of the phylogenetic reconstruction was to give an overview over the different examined clades with our choice of fungi as well as to visually summarize our findings in an appealing way (see Figure 1).

### Ethanol-Based Culturing

**FIGURE 2:**
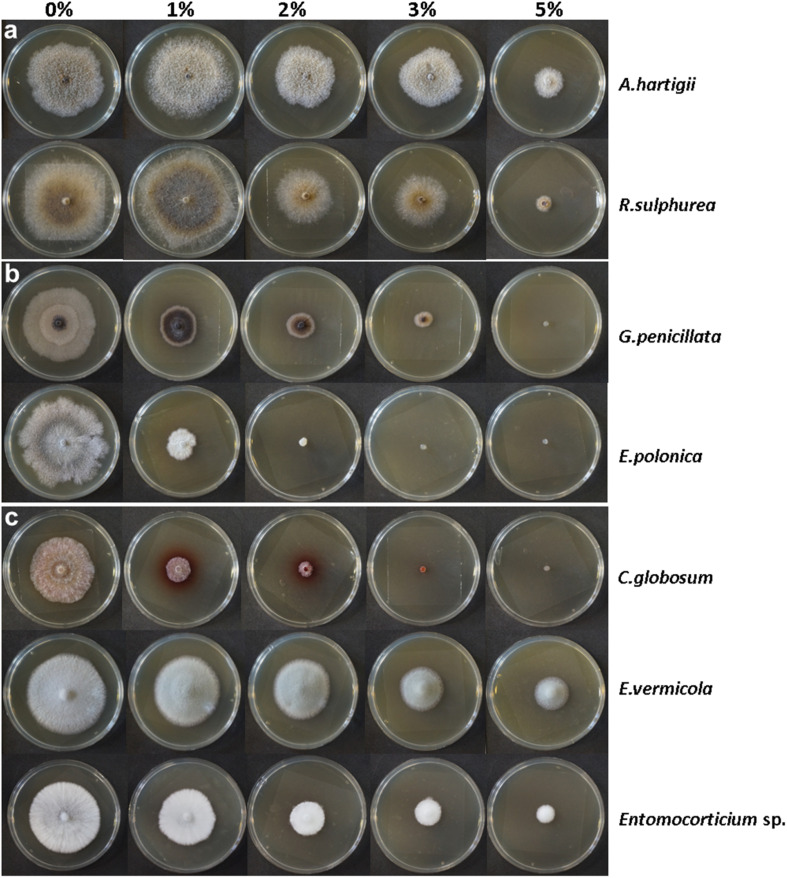
Representative images of seven studied fungi growing on MEA treated with five investigated ethanol concentrations (0, 1, 2, 3, and 5% vol/vol). **(a)**: ambrosia beetle fungi = *Ambrosiella hartigii*, *Raffaelea sulphurea*; **(b)**: bark beetle fungi = *Grosmannia penicillata*, *Endoconidiophora polonica*; **(c)**: pathogens/free-living = *Chaetomium globosum*, *Esteya vermicola*, *Entomocorticium* sp. For incubation times and further information of each individual strain see Supplementary Table 3. The five remaining fungal species are displayed in Supplemantary Figure 1.

We examined the influence of five ethanol (EtOH) concentrations in MEA culturing media (0, 1, 2, 3, 5 vol/vol) on biomass, area and density of 12 fungal strains. The methods closely followed those reported in Ranger et al. (2018). Ethanol (99.8%, Sigma-Aldrich, Germany) was added to the MEA media at around 55°C (just before it solidified) to reduce its evaporation. Immediately after the media had cooled down in the petri dishes, we added a sterile cellophane membrane (6 × 6 cm, NeoLab, Germany) and a plug of the pre-cultured fungal mycelium (Ø 3 mm, using a cork borer) to the center of each plate. We used eight replicates per fungus for each of the five EtOH treatments, for a total of 40 plates per fungus, which were sealed with parafilm and subsequently incubated in dark at 25°C and 60% RH. Images of the colonies were taken every second day (Sony alpha 5000, 12 × in macro mode) through the closed lid of the petri dish. Only at the last day of the experiment the lid was removed before taking the picture (see Figure 2 and Supplementary Figure 1). All images were analyzed for surface area using ImageJ software (version 1.52a). The experiment was terminated for each fungal species separately and as soon as one replicate (independent of the EtOH treatment) reached an edge of the cellophane membrane. For individual incubation times see Supplementary Table 3. If single replicates were contaminated, we excluded them from our analyses (Supplementary Table 3). After termination of the experiment and taking of the picture, the entire fungal biomass was collected from each cellophane membrane, dried individually at 50°C for up to 14 days (dry biomass) in a drying oven and was then weighed. To calculate the density (mg/mm^2^) of fungal mycelia, the determined dry weight (mg) was divided by measured surface area (mm^2^).

Effects of EtOH concentration on dry biomass, surface area and density for each fungal species were visualized with ggplot in R (version 1.2.5033) using the package ggplot2 (Wickham, 2016). One-way-ANOVAs (log10 transformed data; Biomass in dependence of ethanol concentration) were conducted to detect differences in the biomass between the 0 and 1%, and the 0 and 2% EtOH treatments for each fungus using the package rcompanion (CRAN.R-project.org/package = rcompanion). We added the received ANOVA output for each examined fungus to the Anova Output.

## Results

### Fungal Biomass in Relation to the Amount of EtOH in the Media

**FIGURE 3:**
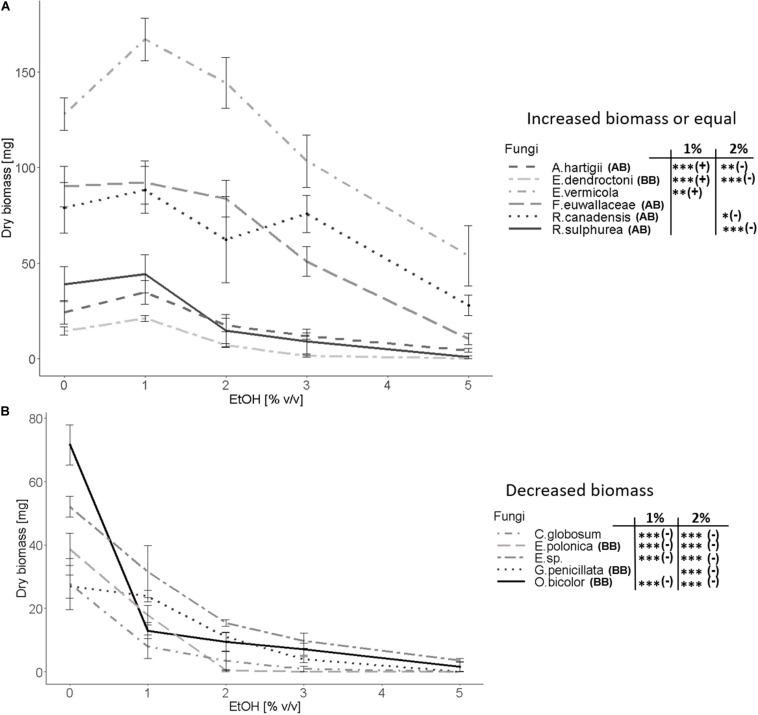
Fungal biomass in mg (dry-weight) as a function of the EtOH (% v/v) treatment (0, 1, 2, 3, and 5% vol/vol). Fungi were classified into **(A)** positively affected (*Ambrosiella hartigii*, *Entomocorticium dendroctoni*, *Esteya vermicola*) and not affected (*Raffaelea canadensis*, *R. sulphurea Fusarium euwallaceae*) by the 1% EtOH treatment or **(B)** negatively affected (*Chaetomium globosum*, *Endoconidiophora polonica*, *Entomocorticium sp.* (E.sp.), *Grosmannia penicillata*, *Ophiostoma bicolor*) by the 1% EtOH treatment. With one exception (*E. vermicola*) all species in the first group are nutritional mutualists of bark and ambrosia beetles, whereas all species in the second group are either plant-defenses-detoxifying mutualists of bark beetles, free-living or antagonistic species. (AB) denotes ambrosia beetle associates and (BB) bark beetle associates. Means and standard deviations are given for each EtOH treatment. Significant differences between the 0% and 1% and 2% EtOH treatments are given: *p < 0.05, **p < 0.01, ***p < 0.001. Additionally, we added (+) and (–) to indicate a significant increase, respectively, decrease of fungal biomass. *N* = 8 replicates per fungal species.

Ethanol enriched substrate had a strong effect on the majority of examined fungal species. Three different reactions of tested fungi toward EtOH could be distinguished: (I) Fungal biomass was positively affected (Figure 3A) (II) not affected (Figure 3A) or (III) negatively affected (Figure 3B) by medium containing 1% ethanol in comparison to the absence of ethanol. Fungal species were only associated with pattern I or III, if a significant difference between the 0 and 1% EtOH treatment could be detected. In case we did not detect any significances, fungi were generally associated with pattern II, even though some species showed a slight non-significant increase or decrease of biomass.

Within the Microascales, we observed significantly more fungal biomass at 1% EtOH (pattern I) for the nutritional mutualist of the ambrosia beetle *Anisandrus dispar*, *Ambrosiella hartigii* (*p* = 0.0008, Figure 2a) while the detoxifying mutualist of the bark beetle *Ips typographus*, *Endoconidiophora polonica* (*p* = 2.23e-10, Figure 2b) produced significantly fewer biomass and thus showed pattern III. In the Opiostomatales, the non-beetle-associated, endophyte *Esteya vermicola* (*p* = 0.008, Figure 2c) clearly showed pattern I while there was no significant increase in biomass but a general tolerance toward ethanol (pattern II) for the two nutritional mutualists of the ambrosia beetle *Xyleborinus saxesenii*, *Raffaelea sulphurea* (*p* = 0.65, Figure 2a) and *R. canadensis* (*p* = 0.35, Supplementary Figure 1B), (mean biomass for both was higher at 1% EtOH, but not statistically significant). The tree-defenses-detoxifying mutualist of *I. typographus*, *Grosmannia penicillata* (*p* = 0.154, Figure 2b) showed a tolerance toward the 1% EtOH treatment, even though mean biomass decreased with increasing EtOH concentrations (no significance at 1% EtOH (pattern II), but rather similar to pattern III). Another symbiont with unknown role in *I. typographus, Ophiostoma bicolor* (*p* = 4.01e-11, Supplementary Figure 1C), was highly negatively affected by EtOH (pattern III). The only Sordariales, the antagonistic bark and ambrosia beetle symbiont *Chaetomium globosum* (*p* = 1.83e-06, Figure 2c) produced significantly fewer biomass (pattern III). Within the Russulales, the supposedly free-living, endophytic *Entomocorticium* sp. (isolated from *Trypodendron lineatum*) shows pattern III (*p* = 1.73e-06, Figure 2c), while the nutritional mutualist of the bark beetle *Dendroctonus ponderosae, Entomocorticium dendroctoni* shows pattern I (*p* = 0.00003, Supplementary Figure 1C). In the Hypocreales, the only fungal species which was tolerating EtOH even up to 2% (pattern II) is the nutritional mutualist of the ambrosia beetle *Euwallacea fornicatus*, *Fusarium euwallaceae* (*p* = 0.82, Supplementary Figure 1B). Within the same group, the generally entomopathogenic fungus *Beauveria bassiana* (Supplementary Figure 1A) showed an enormous standard deviation due to its abundant sporulation, which made it impossible to compare the biomass of this species at different ethanol concentrations as spores easily spread all over the plate. Its growth patterns should be treated with care and are thus only presented in Supplementary Table 3 and Supplementary Figure 1A. All examined fungi produced less biomass above 2 to 3% EtOH compared to the 0% EtOH treatment. This effect was particularly present in the bark beetle symbionts (*E. polonica*, *O. bicolor*, *G. penicillata*, *E. dendroctoni*), the antagonist *C. globosum* and the endophyte *Entomocorticium* sp., while the remaining ambrosia beetle mutualists were less strongly affected. The strongest effect could be observed at the 5% EtOH media, on which only ambrosia beetle mutualists as well as the free-living endophyte *E. vermicola* produced substantial amounts of biomass (see Figure 2, Supplementary Figure 1 and Supplementary Table 3). Results from fungal surface area as well as the calculated density in relation to the amount of EtOH in the media can be found in the Supplementary Results.

## Discussion

Our findings showed that the examined obligate and nutritional ambrosia beetle mutualists (*A. hartigii*, *R. canadensis*, *R. sulphurea*) as well as the obligate and nutritional bark beetle mutualist (*E. dendroctoni*) benefit or at least are not harmed (*F. euwallaceae*) by the presence of low (1–2%) concentrations of ethanol. Most fascinating, this tolerance of ethanol by mutualistic fungi is apparently not restricted to a specific lineage of ambrosia beetle fungi (Figure 1), but must have evolved convergently in different orders of fungi, such as the ascomycete Microascales, Ophiostomatales and Hypocreales as well as the basidiomycete Russulales. Interestingly, the majority of non-nutritional, plant-defenses-detoxifying bark beetle fungi (*E. polonica*, *G. penicillata*, *O. bicolor*) and the fungal antagonist of bark and ambrosia beetles *C. globosum* strongly decrease in biomass in the presence of ethanol. Thus, we confirm and further expand the findings of Ranger et al. (2018) (that were based on just a handful of fungi) to some more lineages of independently evolved bark and ambrosia beetle symbionts with beneficial to antagonistic roles. Our findings show that non-nutritional mutualists of bark beetles (which have never been investigated for their ethanol tolerance) are sensitive to even small amounts of ethanol. This is fascinating because at least in the Ophiostomatales and Microascales these fungi are ancestral to ambrosia beetle mutualists, suggesting that ethanol tolerance convergently evolved repeatedly along with the xylem-boring and fungus-farming habit of ambrosia beetles. Interestingly, all symbionts of bark and ambrosia beetles can tolerate or benefit by the presence of 1–2% ethanol if they are nutritionally beneficial to their obligately dependent beetle host. This is particularly interesting because we have pairs of ethanol benefiting/tolerant vs. sensitive fungal species in almost every fungal order that we tested. It indicates that ethanol tolerance is a derived trait in bark and ambrosia beetle nutritional mutualists that are transmitted in mycetangia and actively farmed by their hosts. Furthermore, the ethanol preference means that these fungi (as well as their hosts) are adapted to colonize stressed or recently dead trees (Moeck, 1970; Miller and Rabaglia, 2009; Ranger et al., 2011, 2013, 2015, 2018; Reding et al., 2011).

While the group of bark and ambrosia beetles are best known by their tree-killing species, the majority of species actually colonizes highly stressed or recently dead host trees (Batra, 1963; Beaver et al., 1989; Kühnholz et al., 2001; Hulcr and Dunn, 2011; Kirkendall et al., 2015; Ranger et al., 2015). On the tree, they differ in the localization of their tunnels – xylem for ambrosia and phloem for bark beetles – and their association with fungi that are typically nutritionally important for ambrosia beetles and detoxify (or induce) tree defenses for bark beetles (but see exceptions in some *Dendroctonus* and *Ips* spp.) (Batra, 1963, 1967; Francke-Grosmann, 1963, 1966; Whitney et al., 1987; Kirkendall et al., 2015; Hulcr and Stelinski, 2017; Vissa and Hofstetter, 2017; Kandasamy et al., 2019). As we discussed above, ambrosia beetles are generally associated with ethanol tolerant or benefiting fungi, whereas bark beetle fungi are typically sensitive to ethanol. This is also reflected by the attraction to ethanol during tree-host finding only of ambrosia beetles, but not of bark beetles. Studies show that ethanol can be found in both xylem and phloem tissue (Kimmerer and Stringer, 1988; MacDonald and Kimmerer, 1991; Kelsey et al., 2014) and ethanol is even enriched in the plant tissues during bark beetle attacks (Kelsey et al., 2014). However, it is unclear if phloem of dying trees is generally lower in ethanol (e.g., due to evaporation) or if bark and ambrosia beetles differ in host substrate preferences. Also, it is possible that both bark and ambrosia beetles have to be exposed to ethanol in their breeding substrate, but that bark beetle symbionts (e.g., bacteria) are capable of detoxifying the phloem. A variety of yeasts, bacteria and filamentous fungi have been isolated from bark beetles (Six, 2013) and many of them have detoxifying capabilities (Dowd, 1989, 1992; Skrodenyte-Arbaciauskiene et al., 2006; Hammerbacher et al., 2013; Um et al., 2013; Ramadhar et al., 2014; Wadke et al., 2016; Zhao et al., 2019).

All fungi showing a tolerance (*R. sulphurea*, *R. canadensis*, *F. euwallacea, G. penicillata*) or even an increase in biomass at 1% ethanol (*E. vermicola*, *A. hartigii*, *E. dendroctoni*, Figure 3A) might indicate an elevated activity of alcohol dehydrogenases (ADHs; referring to the ADH experiment in Ranger et al., 2018). These enzymes allow organisms to detoxify ethanol and even consume it as a carbon source, which can lead to an increase in biomass in the presence of EtOH, as we found in some species (significant increase: *E. vermicola*, *A. hartigii*, *E. dendroctoni*; not significant, but higher mean biomass: *R. canadensis*, *R. sulphurea*, F. euwallaceae). The presence and high activity of ADHs has already been supported by Ranger et al. (2018) for the ambrosia beetle fungal mutualists *Ambrosiella grosmanniae* and *Raffaelea canadensis*, while ADHs were only poorly present in *Ambrosiella roeperi* and not detectable within the yeast-like fungus *Ascoidea* sp. and the fungal antagonist *Aspergillus* sp. Ranger et al. (2018) found a significant increase in biomass at 1% ethanol for *R. canadensis* that we did not find.

Our findings regarding the sensitivity of plant-defenses-detoxifying mutualists of bark beetles toward ethanol (pattern III) in contrast to the insensitivity of nutritional mutualists of bark and ambrosia beetles (pattern I and II) should be interpreted with care, however. Here, we tested only a small fraction of fungi, with only one nutritional mutualist of a bark beetle and no plant-defenses-detoxifying mutualists of ambrosia beetles. Therefore, it is currently unclear whether the ethanol sensitivity that is certainly more common in bark beetle symbionts is related to the phloem habitat or the beneficial physiology of the fungi to the beetles.

In the Hypocreales, the fourth lineage of ambrosia beetle fungal mutualists, *F. euwallaceae*, was neither facilitated nor harmed by EtOH up to 2% relative to the control (Figure 3A). This indicates that this fungus can tolerate EtOH, which is already known for the genus *Fusarium*; for example, *F. oxysporum* is known to produce ethanol (Ueng and Gong, 1982; Christakopoulos et al., 1989). This might indicate that the insensitivity toward ethanol had been present at the origin of the ambrosia beetle-fungus mutualism in the Hypocreales and did not evolve anew as hypothesized for the other lineages (see above). The endophytic ascomycete *E. vermicola* (Ophiostomatales) (Figure 3A) can also metabolize ethanol, similar to ambrosia beetle fungal mutualists. This is especially interesting as this fungus is not associated with any beetle (Liou et al., 1999), but is phylogenetically closely related to the ambrosia beetle fungal mutualist *R. sulphurea* (Figure 1). This could mean that ethanol tolerance is present in some free-living fungi, or even that *E. vermicola* has been previously associated with beetles. Close examination of the phylogeny of the relatives of *E. vermicola* and *R. sulphurea* and their EtOH sensitivities have to be conducted to answer this question.

Quite unique for filamentous fungal species is the high tolerance (reflected by the ability to produce some biomass) of some ambrosia beetle mutualists (*A. hartigii*, *R. canadensis*, and *F. euwallaceae*) as well as some free-living fungi (*E. vermicola* and *Entomocorticium* sp.) toward even the highest tested EtOH concentration (5%). (Figures 2a,c and Supplementary Figure 1B). This tolerance is close to the naturally occurring, wild-type strains of yeasts (up to 6% ethanol by certain species in the genus *Saccharomyces*, *Candida*, *Fabospora*, *Kluyveromyces*, *Kloeckera*) and of specific filamentous fungi (3–7% by certain species in the genus *Rhizopus* and *Fusarium*) used for producing ethanol and above the typical limits of alcohol tolerance in other fungal species (Gao and Fleet, 1988; Singh and Kumar, 1991; Singh et al., 1992; Skory et al., 1997; Banat et al., 1998; Pina et al., 2004; Benjaphokee et al., 2012; Ferreira et al., 2014; Lam et al., 2014; Paschos et al., 2015; Ruchala et al., 2020). In biotechnology, ethanol tolerance and production of fungi can be increased by culture dependent methods (e.g., pH, temperature, medium, carbon-source), genetic modifications and artificial selection (e.g., Gao and Fleet, 1988; Skory et al., 1997; Pina et al., 2004; Benjaphokee et al., 2012; Lam et al., 2014; Ruchala et al., 2020). The extraordinary tolerance of some of our tested fungi in combination with the known ethanol production of ambrosia beetle fungi makes them quite interesting for biotechnological purposes (i.e., second-generation biofuels made from plant biomass). Moreover, as we show here, their tolerance toward ethanol evolved apparently several times independently in unrelated fungal lineages, so it might be fruitful for biotechnological purposes to investigate whether the tolerance is always enabled by the same physiological mechanisms.

Here, we show that not only specific ambrosia beetles, but many ambrosia beetle species and even a *Dendroctonus* bark beetle rely on fungal partners that detoxify and metabolize ethanol. This competitive ability of their mutualistic partners may allow the beetles to indirectly select their partners by biological screening through the ethanol-containing substrate they choose for boring their galleries (Scheuring and Yu, 2012; Ranger et al., 2018). By contrast, most non-nutritional bark beetle fungal mutualists lack the ability to tolerate ethanol. Further studies are necessary to test whether ethanol is generally absent in most of the phloem colonized by bark beetles or is degraded by other symbionts in the microbiome of the beetles.

## Data Availability Statement

All obtained fungal sequences were uploaded to the NCBI GenBank database (the accession numbers are included in Supplementary Table 1).

## Author Contributions

ML and PB designed the study and wrote the manuscript. ML isolated and sequenced all examined fungi, analyzed data, did statistics, plotted results, and constructed the phylogenetic tree. MB and ML did the ethanol-based culturing. All authors contributed to the article and approved the submitted version.

## Conflict of Interest

The authors declare that the research was conducted in the absence of any commercial or financial relationships that could be construed as a potential conflict of interest.

## Footnotes

1 hiv.lanl.gov/content/sequence/findmodel/findmodel.html

2 http://tree.bio.ed.ac.uk/software/figtree

## Supplementary Material

Supplementary Figure 1 Representative images of five studied fungi growing on MEA treated with five investigated ethanol concentrations (0, 1, 2, 3, and 5% vol/vol). **(A)**: entomopathogens = *Beauveria bassiana*; **(B)**: ambrosia beetle fungi = *Raffaelea canadensis*, *Fusarium euwallaceae*; **(C)**: bark beetle fungi = *Entomocorticium dendroctoni*, *Ophiostoma bicolor*. For incubation times and further information on each individual strain see Supplementary Table 3. The seven other fungal species are displayed in Figure 1.

Supplementary Figure 2 **(A)** = Surface area in mm^2^ and **(B)** = density in mg/mm^2^ based on EtOH treatment (0, 1, 2, 3, 5 vol/vol) of fungi positively affected (*Ambrosiella hartigii*, *Entomocorticium dendroctoni*, *Esteya vermicola*) and not affected (*Raffaelea canadensis*, *R. sulphurea, Fusarium euwallaceae*) in dry weight (see Figure 3) by the 1% compared to the 0% EtOH treatment. Means and standard deviations are given. *N* = 8 replicates per fungal species.

Supplementary Figure 3 **(A)** = Surface area in mm^2^ and **(B)** = density in mg/mm^2^ based on the EtOH treatments (0, 1, 2, 3, 5%) of fungi negatively affected (*Chaetomium globosum*, *Endoconidiophora polonica*, *Entomocorticium* sp. (E.sp.), *Grosmannia penicillata*, *Ophiostoma bicolor*) in dry weight (see Figure 3) by the 1% compared to the 0% EtOH treatment. Means and standard deviations are given. *N* = 8 replicates per fungal species.
