# Supplementary figures and images for: Ethanol-Enriched Substrate Facilitates Ambrosia Beetle Fungi, but Inhibits Their Pathogens and Fungal Symbionts of Bark Beetles
Source: Front Microbiol. 2021 Jan 13;11:590111. doi: 10.3389/fmicb.2020.590111 (PMC7838545; doi:10.3389/fmicb.2020.590111)

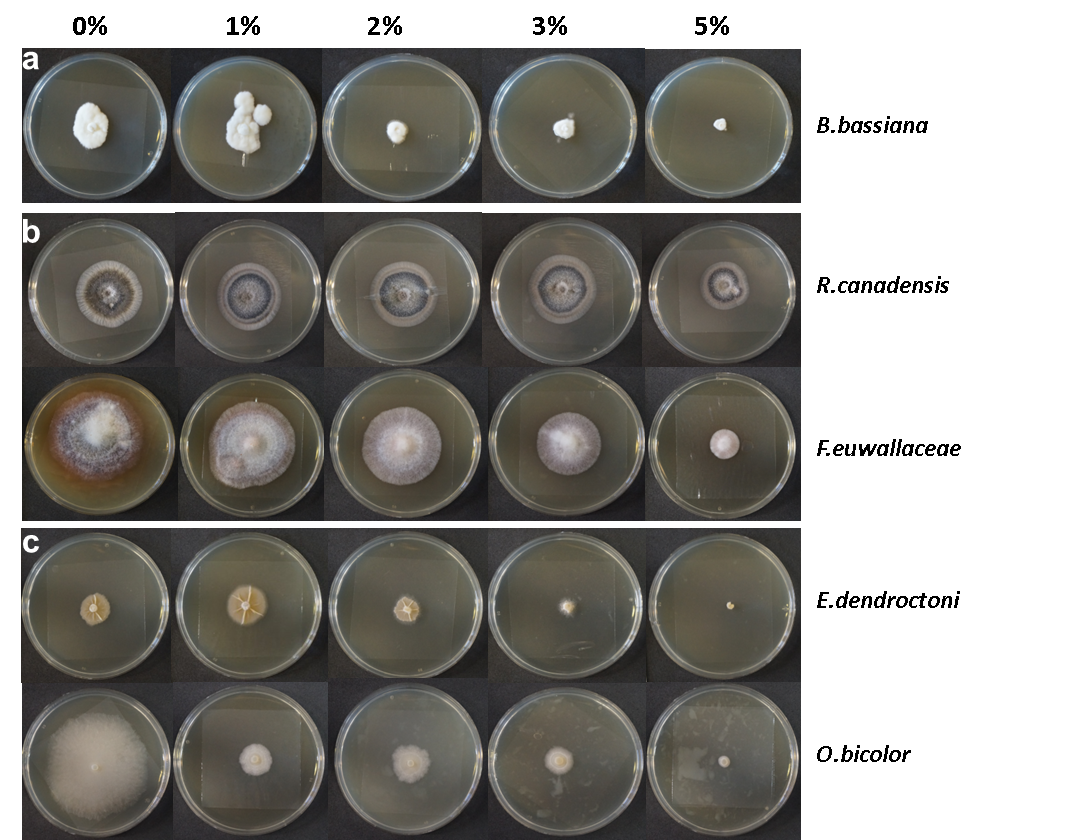

Supplement: Supplementary Figure 1 — Representative images of five studied fungi growing on MEA treated with five investigated ethanol concentrations (0, 1, 2, 3, and 5% vol/vol). (A): entomopathogens = Beauveria bassiana; (B): ambrosia beetle fungi = Raffaelea canadensis, Fusarium euwallaceae; (C): bark beetle fungi = Entomocorticium dendroctoni, Ophiostoma bicolor. For incubation times and further information on each individual strain see Supplementary Table 3. The seven other fungal species are displayed in Figure 1. [file Image_1.TIF]

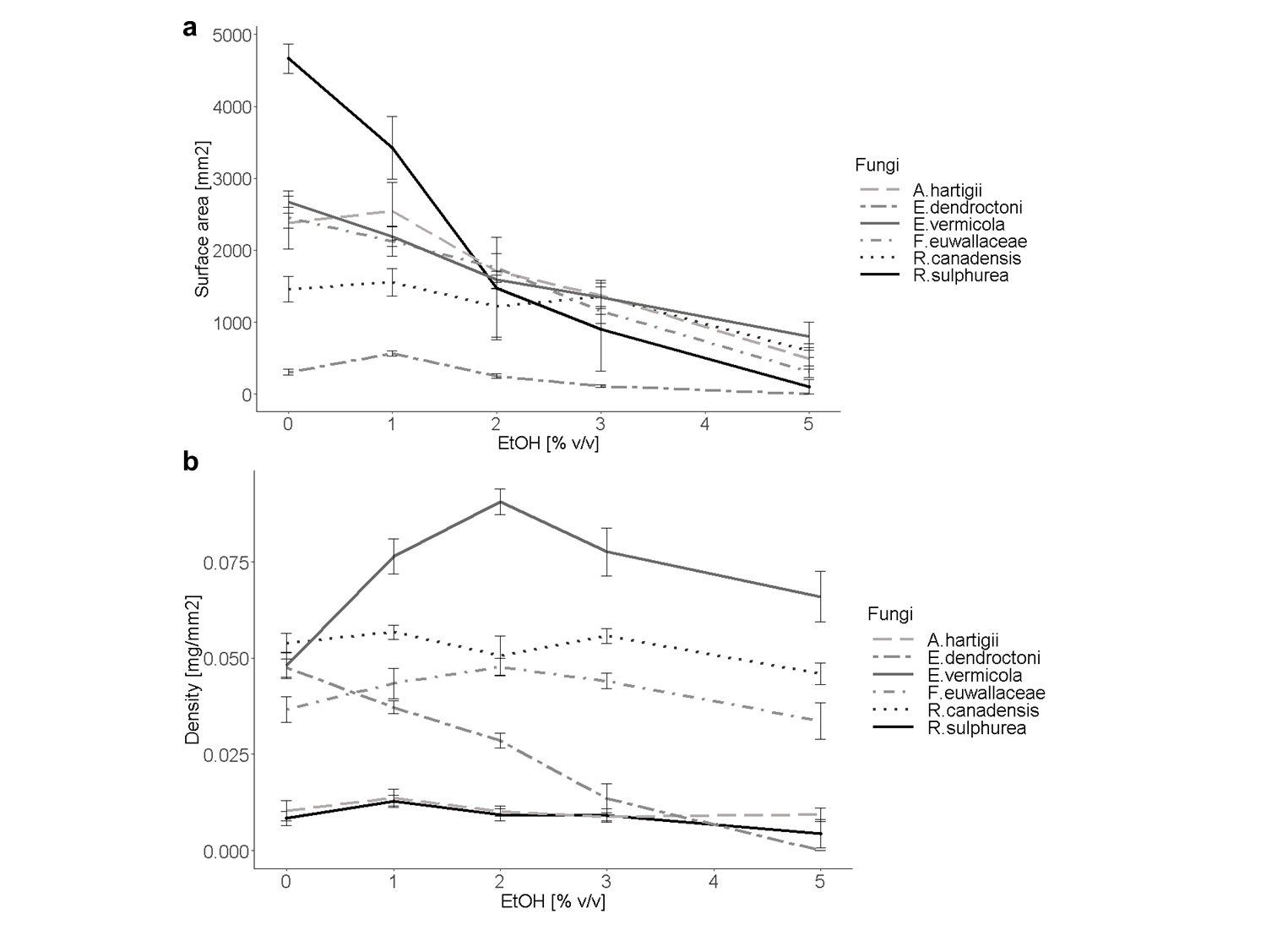

Supplement: Supplementary Figure 2 — (A) = Surface area in mm2 and (B) = density in mg/mm2 based on EtOH treatment (0, 1, 2, 3, 5 vol/vol) of fungi positively affected (Ambrosiella hartigii, Entomocorticium dendroctoni, Esteya vermicola) and not affected (Raffaelea canadensis, R. sulphurea, Fusarium euwallaceae) in dry weight (see Figure 3) by the 1% compared to the 0% EtOH treatment. Means and standard deviations are given. N = 8 replicates per fungal species. [file Image_2.TIF]

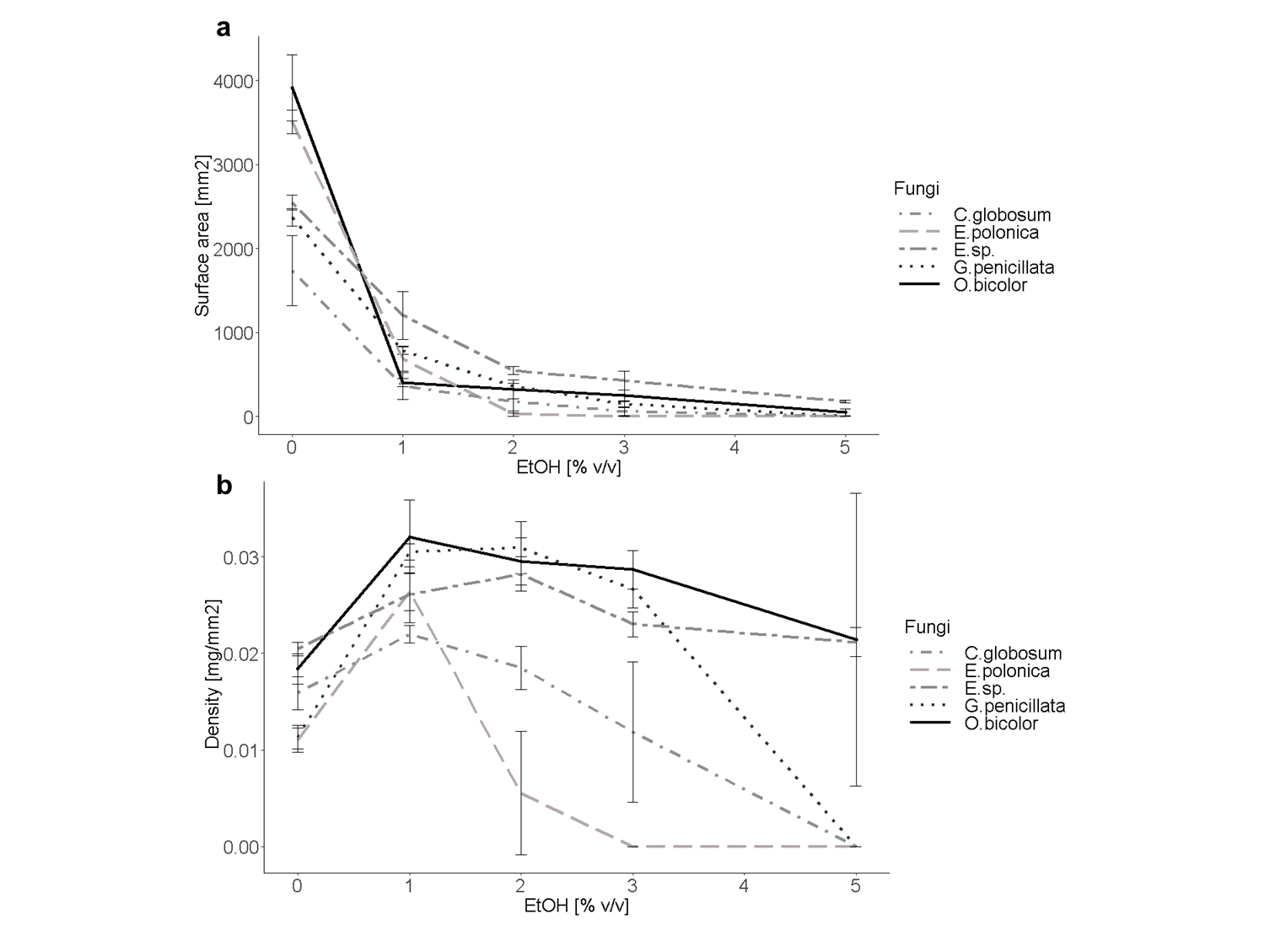

Supplement: Supplementary Figure 3 — (A) = Surface area in mm2 and (B) = density in mg/mm2 based on the EtOH treatments (0, 1, 2, 3, 5%) of fungi negatively affected (Chaetomium globosum, Endoconidiophora polonica, Entomocorticium sp. (E.sp.), Grosmannia penicillata, Ophiostoma bicolor) in dry weight (see Figure 3) by the 1% compared to the 0% EtOH treatment. Means and standard deviations are given. N = 8 replicates per fungal species. [file Image_3.TIF]
