# Supplementary material for: Ethanol-Enriched Substrate Facilitates Ambrosia Beetle Fungi, but Inhibits Their Pathogens and Fungal Symbionts of Bark Beetles
Source: Front Microbiol. 2021 Jan 13;11:590111. doi: 10.3389/fmicb.2020.590111 (PMC7838545; doi:10.3389/fmicb.2020.590111)
Supplement: Supplementary file 4 [file Data_Sheet_1.docx]

**Results**

**Fungal surface area and density in relation to the amount of EtOH in the media**

(**I** & **II**) The surface area at 1% EtOH in the media slightly increased for *E.dendroctoni*, *A.hartigii* and *R.canadensis* relative to the 0% treatment, while it decreased relative to the control for the former two at >1% EtOH and for *R.canadensis at* >2% EtOH. For *E.vermicola*, *F.euwallaceae* and *R.sulphurea*, we observed a constant decrease in surface area (see Suppl. Fig. 2a). *R.sulphurea* is quite distinct regarding surface area when compared to the other ambrosia beetle fungi due to a higher production of aerial mycelium. *A.hartigii*, *R.sulphurea*, and *R.canadensis* showed a higher mycelial density at the 1% treatment and *E.vermicola* and *F.euwallaceae* at the 2% treatment, while it decreased for *E.dendroctoni* in the presence of ethanol (see Suppl. Fig. 2b). (**III**) Except *E.dendroctoni*, all bark beetle fungi (*E.polonica*, *G.penicillata*, *O.bicolor*), the antagonistic fungus *C.globosum* and the free-living *Entomocorticium* sp. were strongly inhibited by the presence of ethanol (Suppl. Fig. 3a). *O.bicolor*, *G.penicillata*, *E.polonica* and *C.globosum* showed their highest mycelial densities at the 1% treatment while *Entomocorticium* sp. had its peak at 2% EtOH. The density decreased for all species >2% EtOH.
