# Supplementary material for: Ethanol-Enriched Substrate Facilitates Ambrosia Beetle Fungi, but Inhibits Their Pathogens and Fungal Symbionts of Bark Beetles
Source: Front Microbiol. 2021 Jan 13;11:590111. doi: 10.3389/fmicb.2020.590111 (PMC7838545; doi:10.3389/fmicb.2020.590111)
Supplement: Supplementary file 5 [file Data_Sheet_2.docx]

R. sulphurea:

Residuals:

Min 1Q Median 3Q Max

-0.59317 -0.17456 0.02722 0.15574 0.37112

Coefficients:

Estimate Std. Error t value Pr(>|t|)

(Intercept) 1.59038 0.08731 18.216 < 2e-16 ***

EtOH1% 0.05542 0.12347 0.449 0.656420

EtOH2% -0.47363 0.12781 -3.706 0.000746 ***

EtOH3% -0.71846 0.12347 -5.819 1.48e-06 ***

EtOH5% -1.38287 0.12347 -11.200 5.93e-13 ***

---

Signif. codes: 0 ‘***’ 0.001 ‘**’ 0.01 ‘*’ 0.05 ‘.’ 0.1 ‘ ’ 1

Residual standard error: 0.2469 on 34 degrees of freedom

Multiple R-squared: 0.8425, Adjusted R-squared: 0.8239

F-statistic: 45.46 on 4 and 34 DF, p-value: 3.467e-13

E. vermicola:

Residuals:

Min 1Q Median 3Q Max

-0.227880 -0.015408 0.009018 0.033529 0.166957

Coefficients:

Estimate Std. Error t value Pr(>|t|)

(Intercept) 2.10961 0.02786 75.711 < 2e-16 ***

EtOH1% 0.11462 0.04079 2.810 0.00815 **

EtOH2% 0.05086 0.03941 1.291 0.20550

EtOH3% -0.09553 0.03941 -2.424 0.02080 *

EtOH5% -0.39177 0.03941 -9.942 1.35e-11 ***

---

Signif. codes: 0 ‘***’ 0.001 ‘**’ 0.01 ‘*’ 0.05 ‘.’ 0.1 ‘ ’ 1

Residual standard error: 0.07881 on 34 degrees of freedom

Multiple R-squared: 0.8533, Adjusted R-squared: 0.836

F-statistic: 49.43 on 4 and 34 DF, p-value: 1.049e-13

R. canadensis:

Residuals:

Min 1Q Median 3Q Max

-0.198163 -0.045196 -0.009103 0.054814 0.250392

Coefficients:

Estimate Std. Error t value Pr(>|t|)

(Intercept) 1.89680 0.03503 54.154 < 2e-16 ***

EtOH1% 0.05022 0.05350 0.939 0.3547

EtOH2% -0.12230 0.04953 -2.469 0.0189 *

EtOH3% -0.01539 0.04953 -0.311 0.7579

EtOH5% -0.44346 0.04953 -8.953 2.4e-10 ***

---

Signif. codes: 0 ‘***’ 0.001 ‘**’ 0.01 ‘*’ 0.05 ‘.’ 0.1 ‘ ’ 1

Residual standard error: 0.09907 on 33 degrees of freedom

Multiple R-squared: 0.7892, Adjusted R-squared: 0.7636

F-statistic: 30.88 on 4 and 33 DF, p-value: 9.796e-11

O. bicolor:

Residuals:

Min 1Q Median 3Q Max

-0.33735 -0.06918 0.00452 0.06409 0.41083

Coefficients:

Estimate Std. Error t value Pr(>|t|)

(Intercept) 1.85935 0.05402 34.421 < 2e-16 ***

EtOH1% -0.71925 0.07639 -9.415 4.01e-11 ***

EtOH2% -0.86437 0.07639 -11.315 3.04e-13 ***

EtOH3% -0.96335 0.07639 -12.610 1.42e-14 ***

EtOH5% -1.52199 0.07639 -19.923 < 2e-16 ***

---

Signif. codes: 0 ‘***’ 0.001 ‘**’ 0.01 ‘*’ 0.05 ‘.’ 0.1 ‘ ’ 1

Residual standard error: 0.1528 on 35 degrees of freedom

Multiple R-squared: 0.9214, Adjusted R-squared: 0.9124

F-statistic: 102.6 on 4 and 35 DF, p-value: < 2.2e-16

G. penicillata:

Residuals:

Min 1Q Median 3Q Max

-0.247470 -0.006735 0.000000 0.034153 0.099318

Coefficients:

Estimate Std. Error t value Pr(>|t|)

(Intercept) 1.44182 0.02236 64.484 < 2e-16 ***

EtOH1% -0.04603 0.03162 -1.456 0.154

EtOH2% -0.36084 0.03162 -11.412 2.4e-13 ***

EtOH3% -0.76299 0.03162 -24.129 < 2e-16 ***

EtOH5% -1.44182 0.03162 -45.597 < 2e-16 ***

---

Signif. codes: 0 ‘***’ 0.001 ‘**’ 0.01 ‘*’ 0.05 ‘.’ 0.1 ‘ ’ 1

Residual standard error: 0.06324 on 35 degrees of freedom

Multiple R-squared: 0.9879, Adjusted R-squared: 0.9865

F-statistic: 714.7 on 4 and 35 DF, p-value: < 2.2e-16

E. polonica:

Residuals:

Min 1Q Median 3Q Max

-0.137034 -0.015512 0.000000 0.005889 0.249542

Coefficients:

Estimate Std. Error t value Pr(>|t|)

(Intercept) 1.59529 0.02586 61.70 < 2e-16 ***

EtOH1% -0.32472 0.03657 -8.88 2.23e-10 ***

EtOH2% -1.48311 0.03785 -39.18 < 2e-16 ***

EtOH3% -1.59529 0.03657 -43.63 < 2e-16 ***

EtOH5% -1.59529 0.03657 -43.63 < 2e-16 ***

---

Signif. codes: 0 ‘***’ 0.001 ‘**’ 0.01 ‘*’ 0.05 ‘.’ 0.1 ‘ ’ 1

Residual standard error: 0.07313 on 34 degrees of freedom

Multiple R-squared: 0.9905, Adjusted R-squared: 0.9894

F-statistic: 885.6 on 4 and 34 DF, p-value: < 2.2e-16

1. hartigii:

Residuals:

Min 1Q Median 3Q Max

-0.11504 -0.06689 -0.01110 0.04453 0.17740

Coefficients:

Estimate Std. Error t value Pr(>|t|)

(Intercept) 1.39197 0.02949 47.208 < 2e-16 ***

EtOH1% 0.15282 0.04170 3.665 0.000813 ***

EtOH2% -0.13066 0.04170 -3.133 0.003485 **

EtOH3% -0.28282 0.04170 -6.782 7.31e-08 ***

EtOH5% -0.66471 0.04170 -15.941 < 2e-16 ***

---

Signif. codes: 0 ‘***’ 0.001 ‘**’ 0.01 ‘*’ 0.05 ‘.’ 0.1 ‘ ’ 1

Residual standard error: 0.0834 on 35 degrees of freedom

Multiple R-squared: 0.9278, Adjusted R-squared: 0.9195

F-statistic: 112.4 on 4 and 35 DF, p-value: < 2.2e-16

1. globosum:

Residuals:

Min 1Q Median 3Q Max

-0.39098 -0.09342 -0.00212 0.01546 0.44153

Coefficients:

Estimate Std. Error t value Pr(>|t|)

(Intercept) 1.44329 0.06367 22.667 < 2e-16 ***

EtOH1% -0.51478 0.09005 -5.717 1.83e-06 ***

EtOH2% -0.87622 0.09005 -9.730 1.72e-11 ***

EtOH3% -1.21169 0.09005 -13.456 2.13e-15 ***

EtOH5% -1.44329 0.09005 -16.028 < 2e-16 ***

---

Signif. codes: 0 ‘***’ 0.001 ‘**’ 0.01 ‘*’ 0.05 ‘.’ 0.1 ‘ ’ 1

Residual standard error: 0.1801 on 35 degrees of freedom

Multiple R-squared: 0.9023, Adjusted R-squared: 0.8911

F-statistic: 80.78 on 4 and 35 DF, p-value: < 2.2e-16

E. dendroctoni:

Residuals:

Min 1Q Median 3Q Max

-0.13674 -0.03219 0.00000 0.02048 0.17619

Coefficients:

Estimate Std. Error t value Pr(>|t|)

(Intercept) 1.18543 0.02335 50.760 < 2e-16 ***

EtOH1% 0.15759 0.03303 4.771 3.19e-05 ***

EtOH2% -0.27929 0.03303 -8.456 5.62e-10 ***

EtOH3% -0.79343 0.03303 -24.023 < 2e-16 ***

EtOH5% -1.18543 0.03303 -35.892 < 2e-16 ***

---

Signif. codes: 0 ‘***’ 0.001 ‘**’ 0.01 ‘*’ 0.05 ‘.’ 0.1 ‘ ’ 1

Residual standard error: 0.06605 on 35 degrees of freedom

Multiple R-squared: 0.985, Adjusted R-squared: 0.9833

F-statistic: 575.3 on 4 and 35 DF, p-value: < 2.2e-16

Entomocortcium sp:

Residuals:

Min 1Q Median 3Q Max

-0.30208 -0.03344 -0.00986 0.03383 0.19920

Coefficients:

Estimate Std. Error t value Pr(>|t|)

(Intercept) 1.72451 0.02828 60.980 < 2e-16 ***

EtOH1% -0.22931 0.03999 -5.734 1.73e-06 ***

EtOH2% -0.50973 0.03999 -12.745 1.04e-14 ***

EtOH3% -0.70099 0.03999 -17.528 < 2e-16 ***

EtOH5% -1.05138 0.03999 -26.289 < 2e-16 ***

---

Signif. codes: 0 ‘***’ 0.001 ‘**’ 0.01 ‘*’ 0.05 ‘.’ 0.1 ‘ ’ 1

Residual standard error: 0.07999 on 35 degrees of freedom

Multiple R-squared: 0.9598, Adjusted R-squared: 0.9552

F-statistic: 208.7 on 4 and 35 DF, p-value: < 2.2e-16

F. euwallaceae:

Residuals:

Min 1Q Median 3Q Max

-0.34761 -0.04008 0.00662 0.04323 0.17438

Coefficients:

Estimate Std. Error t value Pr(>|t|)

(Intercept) 1.956218 0.030550 64.033 < 2e-16 ***

EtOH1% 0.009399 0.043205 0.218 0.829

EtOH2% -0.030967 0.043205 -0.717 0.478

EtOH3% -0.245151 0.043205 -5.674 2.08e-06 ***

EtOH5% -0.918412 0.043205 -21.257 < 2e-16 ***

---

Signif. codes: 0 ‘***’ 0.001 ‘**’ 0.01 ‘*’ 0.05 ‘.’ 0.1 ‘ ’ 1

Residual standard error: 0.08641 on 35 degrees of freedom

Multiple R-squared: 0.9502, Adjusted R-squared: 0.9445

F-statistic: 167.1 on 4 and 35 DF, p-value: < 2.2e-16
