# Supplementary material for: Ethanol-Enriched Substrate Facilitates Ambrosia Beetle Fungi, but Inhibits Their Pathogens and Fungal Symbionts of Bark Beetles
Source: Front Microbiol. 2021 Jan 13;11:590111. doi: 10.3389/fmicb.2020.590111 (PMC7838545; doi:10.3389/fmicb.2020.590111)
Supplement: Supplementary file 6 [file Data_Sheet_3.docx]

**Supplementary Material**

**Table S1:** Overview of the origin of the twelve fungal isolates used in this study.

| **Species** | **ID** | **Year of isolation** | **Locality of collection** | **Beetle host** | **Isolated by** | **Sequenced marker** | **Accession numbers** |
| --- | --- | --- | --- | --- | --- | --- | --- |
| *Entomocorticium* sp. | P8 | 2016 | Neuschönau, Bavaria, Germany | *Trypodendron lineatum* | Lehenberger M; Lehenberger et al. 2018 | LSU | MT159435 |
| *Entomocorticium dendroctoni* | P163 | 2018 | Arizona, USA | *Dendroctonus brevicomis* | Lehenberger M; following protocol in Lehenberger et al. 2019 | LSU | MT159436 |
| *Endoconidiophora polonica* | F5 | 2013 | South Karelia, Finland | *Ips typographus* | Unknown | LSU | MT159432 |
| *Ophiostoma bicolor* | P22 | 2004 | Akershus, Norway | *Ips typographus* | Unknown | LSU | MT159431 |
| *Grosmannia penicillata* | 2 | 2006 | Kronoberg county, Sweden | *Ips typographus* | Unknown | LSU | MT159434 |
| *Fusarium euwallaceae* | P170 | 2018 | Israel | *Euwallacea fornicatus* | Mendel Z | LSU | MT159437 |
| *Ambrosiella hartigii* | P137 | 2019 | Würzburg, Bavaria, Germany | *Anisandrus dispar* | Lehenberger M; following protocol in Lehenberger et al. 2019 | ITS | MT840100 |
| *Raffaelea canadensis* | P23 | 2019 | Würzburg, Bavaria, Germany | *Xyleborinus saxesenii* | Lehenberger M; following protocol in Lehenberger et al. 2019 | Beta-tubulin | MT880109 |
| *Raffaelea sulphurea* | P159 | 2019 | Würzburg, Bavaria, Germany | *Xyleborinus saxesenii* | Lehenberger M; following protocol in Lehenberger et al. 2019 | Beta-tubulin | MT880108 |
| *Chaetomium globosum* | P7 | 2018 | Würzburg, Bavaria, Germany | Gallery of *Xyleborinus saxesenii* | Lehenberger M; following protocol in Lehenberger et al. 2019 | ITS | MT840101 |
| *Beauveria bassiana* | P13 | 2019 | Würzburg, Bavaria, Germany | *Ips typographus* | Lehenberger M; following protocol in Lehenberger et al. 2019 | LSU | MT159433 |
| *Esteya vermicola* | CBS156.82 | 1999 | Japan | *Pinus* – dried plant | Liou JY, Shih JY & Tzean | Beta-tubulin | MT880110 |

**Table S2:** Overview of all fungal species used for phylogenetic analysis with the specimen identiﬁcation number (ID) and the corresponding GenBank accession numbers for the LSU marker. * Species from which we sequenced the LSU-region and which we used for the construction of our phylogenetic tree.

| **Annotation** | **ID** | **Accession number** | **Beetle host** | **Reference** |
| --- | --- | --- | --- | --- |
| *Heterobasidion annosum* | CBS 859.87 | **MH873808** | None | Vu et al., 2019 |
| *Peniophora rufa* | B1014 | **MN475831.1** | None | Harrington et al., 2019 Unpublished |
| *P.crassitunicata* | CBS 663.91 | **NG_064161** | None | Vu et al., 2019 |
| *Entomocorticium* sp.* | P8 | **MT159435** | Unknown | Lehenberger et al., 2020, unpublished |
| *E.dendroctoni* * | P163 | **MT159436** | *Dendroctonus brevicornis* | Lehenberger et al., 2020, unpublished |
| *Penicillium commune* | CBS 341.59 | **MH869425.1** | None | Vu et al., 2019 |
| *Raffaelea fusca* | CBS129011 | **MH878043.1** | *Xyleborus glabratus* | Vu et al., 2019 |
| *R.canadensis* | CBS 326.70 | **MH871450.1** | *Xyleborinus saxesenii* | Vu et al., 2019 |
| *R.quercivora* | RA2245 | **LC069313.1** | *Platypus quercivorus* | Torii et al., 2016 |
| *R.montetyi* | PC06.001 | **JF909540.1** | *Platypus cylindrus* | Inacio et al., 2011 Unpublished |
| *R.sulphurea* | C593 | **EU177463.1** | *Xyleborinus saxesenii* | Harrington et al., 2010 |
| *R.amase* | CBS116694 | **EU984295.1** | *Amasa concitatus* | Alamouti et al., 2009 |
| *R.lauricola* | CBS 121567 | **MH877762.1** | *Xyleborus glabratus* | Vu et al., 2019 |
| *R.sulcati* | CBS 806.70 | **NG_064084.1** | *Gnathotrichus sulcatus* | Vu et al., 2019 |
| *R.brunnea* | MAR50.a | **MN012913.1** | *Monarthrum* spp. | Restrepo et al., 2019 Unpublished |
| *R.rapaneae* | CMW40359 | **KT182935** | *Lanurgus* sp. | Musvuugwa et al., 2015 |
| *R.ambrosiae* | C2225 | **EU177453** | *Platypus* spp. | Harrington et al., 2010 |
| *R.santoroi* | CBS 399.67 | **NG_064067** | *Platypus* spp. | Vu et al., 2019 |
| *Raffaelea quercus-mongolicae* | RQM04 | **KF513155** | *Platypus koryoensis* | Park, 2013 Unpublished |
| *Esteya vermicola* | CBS 115803 | **EU668903.1** | None | Wang et al., 2008 |
| *Grosmannia clavigera* | M002 | **GU370280.1** | *Dendroctonus ponderosae* | Roe et al., 2010 |
| *G.aurea* | CMW667 | **DQ294389.1** | *Dendroctonus* spp. | Zipfel et al., 2006 |
| *G.francke-grosmanniae* | CMW2975 | **DQ294395.1** | Unknown | Zipfel et al., 2006 |
| *G.penicillata** | 2 | **MT159434** | *Ips* spp. | Lehenberger et al., 2020, unpublished |
| *Leptographium castellanum* | CBS 128697 | **MH876514.1** | Unknown | Vu et al., 2019 |
| *L.abietinum* | CF19 | **MH084777.1** | *Dendroctonus rufipennis* | Davis et al., 2019 |
| *L.procerum* | CBS 128844 | **MH876524.1** | *Dendroctonus* spp. | Vu et al., 2019 |
| *L.lundbergii* | CBS 128843 | **MH878023** | *Ips* spp. | Vu et al., 2019 |
| *Ophiostoma dryocoetis* | CBS 376.66 | **MH870468.1** | *Dryocoetes confusus* | Vu et al., 2019 |
| *O.piceae* | CBS 180.69 | **MH877705.1** | Unknown | Vu et al., 2019 |
| *O.quercus* | CBS 366.93 | **MH874074.1** | *Platypus* spp. | Vu et al., 2019 |
| *O.piliferum* | CBS 138.33 | **MH866835.1** | Unknown | Vu et al., 2019 |
| *O.bicolor** | P22 | **MT159431** | *Ips* spp. | Lehenberger et al., 2020, unpublished |
| *O.montium* | CMW13221 | **DQ294379.1** | *Dendroctonus ponderosae* | Zipfel et al., 2006 |
| *O.ips* | 9 | **KY946730.1** | *Ips pini* | Pastirčáková et al., 2018 |
| *Sporothrix schenckii* | CBS 359.36 | **KX590890.1** | Unknown | De Beer et al., 2016 |
| *S.stenoceras* | CBS 798.73 | **MH872535.1** | Unknown | Vu et al., 2019 |
| *Neurospora crassa* | ICMP 6360 | **AY681158.1** | None | Cai et al., 2006 |
| *Chaetomium globosum* | CBS 128472 | **MH876396.1** | None | Vu et al., 2019 |
| *Ambrosiella beaveri* | C2749 | **KF646765.2** | *Xylosandrus mutilatus* | Harrington et al. 2014 |
| *A.grosmanniae* | D16 | **MG050696.1** | *Xylosandrus germanus* | Van de Peppel et al., 2018 |
| *A.hartigii* | D17 | **MG050697.1** | *Anisandrus dispar* | Van de Peppel et al., 2018 |
| *A.roeperi* | 14766 | **MG954244.1** | *Xyleborus crassisculus* | Huang et al. 2018 Unpublished |
| *Endoconidiophora polonica** | F5 | **MT159432** | *Ips* spp. | Lehenberger et al., 2020, unpublished |
| *E.pinicola* | CMW29499 | **KM495364.1** | Unknown | De Beer, 2014 Unpublished |
| *Phialophoropsis ferruginea* | CBS 460.82 | **KM495316** | *Trypodendron lineatum* | De Beer, 2014 Unpublished |
| *Baeuveria bassiana** | P13 | **MT159433** | None | Lehenberger et al, 2020, unpublished |
| *Fusarium solani* | CBS490.63 | **AY097316.1** | None | Summerbell and Schroers, 2002 |
| *F.euwallaceae** | P170 | **MT159437** | *Euwallacea fornicatus* | Lehenberger et al., 2020, unpublished |

**Table S3:** Overview of the ethanol assays and the results for dry-biomass, covered area and mycelial density for each fungal isolate. Statistical differences (p) are given only for the dry-biomass and relative to the 0% ethanol treatment: ns - p > 0.05, * - p < 0.05, ** - p < 0.001, *** - p < 0.0001; (+) significant increase, (-) significant decrease.

| **Fungal species** | **Pre-culture-**  **time**  **[d]** | **Incubation-time [d]** | **EtOH-treatment**  **[%]** | **Replicates**  **[N]** | **Dry-biomass**  **Mean**  **[mg]** | **Dry-biomass**  **SD**  **[mg]** | **Dry-biomass p** | **Area**  **mean**  **[mm^2^]** | **Area**  **SD**  **[mm^2^]** | **Density**  **mean**  **[mg/mm^2^]** | **Density**  **SD**  **[mg/mm^2^]** |
| --- | --- | --- | --- | --- | --- | --- | --- | --- | --- | --- | --- |
| *Entomocorticium* sp. | 8 | 7 | 0 | 8 | 52.1 | 3.2 |  | 2547.5 | 84.87 | 0.0204 | 0.0007 |
|  |  |  | 1 | 8 | 31.6 | 8.2 | ***(-) | 1202.6 | 285.22 | 0.0261 | 0.0029 |
|  |  |  | 2 | 8 | 15.4 | 1 | ***(-) | 548.4 | 46.88 | 0.0282 | 0.0018 |
|  |  |  | 3 | 8 | 9.8 | 2.4 | ***(-) | 426.8 | 110.1 | 0.023 | 0.0013 |
|  |  |  | 5 | 8 | 3.7 | 0.5 | ***(-) | 176.1 | 14.3 | 0.0212 | 0.0015 |
| *E. dendroctoni* | 14 | 14 | 0 | 8 | 14.5 | 2.1 |  | 305.2 | 43.228 | 0.0474 | 0.0023 |
|  |  |  | 1 | 8 | 21.1 | 1.6 | ***(+) | 566.4 | 34.099 | 0.0372 | 0.0016 |
|  |  |  | 2 | 8 | 7.1 | 0.8 | ***(-) | 249.1 | 31.526 | 0.0286 | 0.0019 |
|  |  |  | 3 | 8 | 1.5 | 0.7 | ***(-) | 110.6 | 20.78 | 0.0136 | 0.0037 |
|  |  |  | 5 | 8 | 0 | 0 | ***(-) | 0 | 0 | 0 | 0 |
| *Endoconidiophora*  *polonica* | 6 | 6 | 0 | 8 | 38.7 | 5.1 |  | 3509.2 | 139.809 | 0.011 | 0.0013 |
|  |  |  | 1 | 8 | 17.9 | 3 | ***(-) | 686.2 | 150.072 | 0.0264 | 0.002 |
|  |  |  | 2 | 7 | 0.4 | 0.4 | ***(-) | 34.1 | 30.546 | 0.0055 | 0.0064 |
|  |  |  | 3 | 8 | 0 | 0 | ***(-) | 0 | 0 | 0 | 0 |
|  |  |  | 5 | 8 | 0 | 0 | ***(-) | 0 | 0 | 0 | 0 |
| *Ophiostoma bicolor* | 6 | 8 | 0 | 8 | 71.6 | 6.3 |  | 3913.4 | 394.623 | 0.0184 | 0.0016 |
|  |  |  | 1 | 8 | 13 | 2.5 | ***(-) | 405.1 | 49.227 | 0.0321 | 0.0038 |
|  |  |  | 2 | 8 | 9.4 | 3 | ***(-) | 321.3 | 111.264 | 0.0295 | 0.0024 |
|  |  |  | 3 | 8 | 7.1 | 1.9 | ***(-) | 247.4 | 63.886 | 0.0287 | 0.0019 |
|  |  |  | 5 | 8 | 1.6 | 1.5 | ***(-) | 52.4 | 41.180 | 0.0214 | 0.0151 |
| *Grosmannia penicillata* | 6 | 8 | 0 | 8 | 26.9 | 3.6 |  | 2371.5 | 101.369 | 0.0113 | 0.0012 |
|  |  |  | 1 | 8 | 23.9 | 1.8 | ns | 784 | 46.063 | 0.0305 | 0.0008 |
|  |  |  | 2 | 8 | 11.1 | 1.4 | ***(-) | 359.7 | 33.083 | 0.0309 | 0.0027 |
|  |  |  | 3 | 8 | 3.9 | 0.9 | ***(-) | 144.2 | 30.345 | 0.0267 | 0.002 |
|  |  |  | 5 | 8 | 0 | 0 | ***(-) | 0 | 0 | 0 | 0 |
| *Fusarium euwallaceae* | 5 | 7 | 0 | 8 | 90.1 | 10.5 |  | 2453 | 140.9 | 0.0367 | 0.0033 |
|  |  |  | 1 | 8 | 92.1 | 11.2 | ns | 2123.6 | 206.982 | 0.0434 | 0.004 |
|  |  |  | 2 | 8 | 83.7 | 9.7 | ns | 1757.1 | 200.436 | 0.0477 | 0.0023 |
|  |  |  | 3 | 8 | 50.9 | 7.7 | ***(-) | 1158.8 | 180.89 | 0.044 | 0.002 |
|  |  |  | 5 | 8 | 10.4 | 3 | ***(-) | 310.3 | 84.493 | 0.0336 | 0.0048 |
| *Ambrosiella hartigii* | 5 | 4 | 0 | 8 | 24.3 | 6.1 |  | 2382.8 | 366.111 | 0.0103 | 0.0026 |
|  |  |  | 1 | 8 | 34.6 | 6.2 | ***(+) | 2545.2 | 399.027 | 0.0136 | 0.0021 |
|  |  |  | 2 | 8 | 17.6 | 3.5 | **(-) | 1708.6 | 246.778 | 0.0103 | 0.0013 |
|  |  |  | 3 | 8 | 12 | 1.7 | ***(-) | 1367.9 | 180.79 | 0.0088 | 0.001 |
|  |  |  | 5 | 8 | 4.4 | 1 | ***(-) | 495.65 | 146.733 | 0.0093 | 0.0018 |
| *Raffaelea canadensis* | 8 | 14 | 0 | 8 | 78.9 | 13.3 |  | 1456.9 | 178.767 | 0.0538 | 0.0025 |
|  |  |  | 1 | 6 | 88.3 | 88.3 | ns | 1554.4 | 194.69 | 0.0567 | 0.0018 |
|  |  |  | 2 | 8 | 62.3 | 62.3 | *(-) | 1222.3 | 427.909 | 0.0501 | 0.0051 |
|  |  |  | 3 | 8 | 75.7 | 75.7 | ns | 1354.6 | 139.992 | 0.0558 | 0.0019 |
|  |  |  | 5 | 8 | 27.9 | 27.9 | ***(-) | 604.8 | 94.457 | 0.0459 | 0.0028 |
| *R. sulphurea* | 5 | 4 | 0 | 8 | 39 | 9.1 |  | 4666.4 | 202.519 | 0.008 | 0.0019 |
|  |  |  | 1 | 8 | 44.4 | 9.9 | ns | 3425.9 | 438.281 | 0.013 | 0.0015 |
|  |  |  | 2 | 7 | 14.6 | 8.7 | ***(-) | 1469.5 | 712.242 | 0.009 | 0.0015 |
|  |  |  | 3 | 8 | 9.1 | 6.5 | ***(-) | 903.3 | 586.696 | 0.009 | 0.0017 |
|  |  |  | 5 | 8 | 0.8 | 0.8 | ***(-) | 102.7 | 96.904 | 0.004 | 0.0037 |
| *Chaetomium globosum* | 7 | 4 | 0 | 8 | 27.7 | 8 |  | 1739.1 | 418.448 | 0.0159 | 0.0017 |
|  |  |  | 1 | 8 | 8 | 3.7 | ***(-) | 362 | 157.962 | 0.022 | 0.0009 |
|  |  |  | 2 | 8 | 3.5 | 3.1 | ***(-) | 177.5 | 137.486 | 0.019 | 0.0022 |
|  |  |  | 3 | 8 | 0.9 | 0.9 | ***(-) | 58.3 | 48.797 | 0.012 | 0.0073 |
|  |  |  | 5 | 8 | 0 | 0 | ***(-) | 0 | 0 | 0 | 0 |
| *Beauveria bassiana* | 9 | 7 | 0 | 8 | 44 | 20.5 |  | 1030.5 | 888.811 | 0.0541 | 0.0135 |
|  |  |  | 1 | 8 | 51.9 | 14.5 | ns | 1219.5 | 523.438 | 0.0455 | 0.0123 |
|  |  |  | 2 | 8 | 44.5 | 28.1 | ns | 1082.6 | 699.225 | 0.0414 | 0.0056 |
|  |  |  | 3 | 8 | 26.4 | 16.5 | ns | 804.7 | 552.697 | 0.0348 | 0.0068 |
|  |  |  | 5 | 8 | 2.3 | 2 | ***(-) | 98.4 | 94.495 | 0.0264 | 0.0123 |
| *Esteya vermicola* | 9 | 13 | 0 | 8 | 128 | 8.5 |  | 2669.6 | 152.859 | 0.048 | 0.0035 |
|  |  |  | 1 | 7 | 167 | 11.1 | **(+) | 2188.3 | 137.5 | 0.076 | 0.0046 |
|  |  |  | 2 | 8 | 144.3 | 13.3 | ns | 1591.2 | 118.544 | 0.091 | 0.0033 |
|  |  |  | 3 | 8 | 103.3 | 13.6 | *(-) | 1344.3 | 233.985 | 0.078 | 0.0062 |
|  |  |  | 5 | 8 | 53.8 | 15.8 | ***(-) | 803.7 | 194.031 | 0.066 | 0.0066 |

**References**

Alamouti SM, Tsui CKM, Breuil C (2009) Multigene phylogeny of filamentous ambrosia fungi associated with ambrosia and bark beetles. Mycological Research, 113(8):822–835.

Cai L, Jeewon R, Hyde KD (2006) Phylogenetic investigations of *Sordariaceae* based on multiple gene sequences and morphology. Mycological Research, 110(2):137–150.

Davis TS, Stewart JE, Mann A, Bradley C, Hofstetter RW (2019) Evidence for multiple ecological roles of *Leptographium abietinum*, a symbiotic fungus associated with the North American spruce beetle. Fungal Ecology, 38:62–70.

De Beer ZW, Duong TA, Wingfield M (2016) The divorce of *Sporothrix* and *Ophiostoma*: solution to a problematic relationship. Studies in Mycology, 83:165–191.

Harrington T, Aghayeva D, Fraedrich S (2010) New combinations in *Raffaelea*, *Ambrosiella*, and *Hyalorhinocladiella*, and four new species from the redbay ambrosia beetle, *Xyleborus glabratus*. Mycotaxon, 111(1):337–361.

Harrington TC, McNew D, Mayers C, Fraedrich SW, Reed S E (2014) *Ambrosiella roeperi* sp. nov. is the mycangial symbiont of the granulate ambrosia beetle, *Xylosandrus crassiusculus*. Mycologia, 106(4):835–845.

Lehenberger M, J Benz P, Müller J, Biedermann PHW (2018) *Trypodendron domesticum* (Linné) und *Trypodendron lineatum* (Olivier) (Curculionidae; Scolytinae) als potentielle Vektoren von xylobionten und sapro-xylobionten Pilzen. Mitteilungen der Deutschen Gesellschaft für allgemeine und angewandte Entomologie, 21.

Lehenberger M, Biedermann PHW, J Benz P (2019) Molecular identification and enzymatic profiling of *Trypodendron* (Curculionidae: Xyloterini) ambrosia beetle-associated fungi of the genus *Phialophoropsis* (Microascales: Ceratocystidaceae). Fungal Ecology, 38:89-97.

Musvuugwa T, De Beer ZW, Duong TA, Dreyer LL, Oberlander KC, Roets F (2015) New species of ophiostomatales from Scolytinae and Platypodinae beetles in the cape floristic region, including the discovery of the sexual state of *Raffaelea*. Antonie van Leeuwenhoek, 108(4):933–950.

Pastirčáková K, Adamčíková K, Pastirčák M, Zach P, Galko J, Kováč M, Laco J (2018) Two blue-stain fungi colonizing scots pine (*Pinus sylvestris*) trees infested by bark beetles in Slovakia, Central Europe. Biologia, 73(11):1053–1066.

Roe AD, Rice AV, Bromilow SE, Cooke JE, Sperling FA (2010) Multilocus species identification and fungal DNA barcoding: insights from blue stain fungal symbionts of the mountain pine beetle. Molecular Ecology Resources, 10(6):946–959.

Summerbell RC, Schroers HJ (2002) Analysis of phylogenetic relationship of *Cylindrocarpon lichenicola* and *Acremonium falciforme* to the *Fusarium solani* species complex and a review of similarities in the spectrum of opportunistic infections caused by these fungi. Journal of Clinical Microbiology, 40(8):2866–2875.

Torii M, Ito M, Nagao M, Matsuda Y, Ito S (2016) Variations in virulence and hyphal growth of four *Raffaelea quercivora* isolates within *Quercus crispula*. Forest Pathology, 46(3):248–255.

Van de Peppel L, Aanen D, Biedermann PHW (2018) Low intraspecific genetic diversity indicates asexuality and vertical transmission in the fungal cultivars of ambrosia beetles. Fungal Ecology, 32:57–64.

Vu D, Groenewald M, De Vries M, Gehrmann T, Stielow B, Eberhardt U, Al-Hatmi A, Groenewald J, Cardinali G, Houbraken J, et al (2019) Large-scale generation and analysis of filamentous fungal DNA barcodes boosts coverage for kingdom fungi and reveals thresholds for fungal species and higher taxon delimitation. Studies in Mycology, 92:135–154.

Wang CY, Fang ZM, Sun BS, Gu LJ, Zhang KQ, Sung CK (2008) High infectivity of an endoparasitic fungus strain, *Esteya vermicola*, against nematodes. The Journal of Microbiology, 46(4):380.

Zipfel RD, De Beer ZW, Jacobs K, Wingfield BD, Wingfield MJ (2006) Multi-gene phylogenies define *Ceratocystiopsis* and *Grosmannia* distinct from *Ophiostoma*. Studies in Mycology, 55:75–97.
